# Does marital status correlate with the female breast cancer risk? A systematic review and meta-analysis of observational studies

**DOI:** 10.1371/journal.pone.0229899

**Published:** 2020-03-05

**Authors:** Menglin Li, Mei Han, Zijie Chen, Yu Tang, Jie Ma, Zhiying Zhang, Zhenzhu Liu, Ning Zhang, Chongcheng Xi, Jintao Liu, Dong Tian, Xiaoxuan Wang, Xunying Huang, Jingwen Chen, Weiguang Wang, Shuangqing Zhai

**Affiliations:** 1 Department of Traditional Chinese Medicine, Beijing Hospital, National Center of Gerontology, Beijing, P.R.China; 2 Center for Evidence-based Chinese Medicine, Beijing University of Chinese Medicine, Beijing, P.R.China; 3 School of Chinese Medicine, Beijing University of Chinese Medicine, Beijing, P.R.China; 4 Department of Oncology, Chaoyang District Traditional Chinese Medicine Hospital of Beijing City, Beijing, P.R.China; 5 Graduate School, Beijing University of Chinese Medicine, Beijing, P.R.China; 6 Department of Oncology, Dongfang Hospital, Beijing University of Chinese Medicine, Beijing, P.R.China; 7 School of Management, Beijing University of Chinese Medicine, Beijing, P.R.China; Istituto di Ricovero e Cura a Carattere Scientifico Centro di Riferimento Oncologico della Basilicata, ITALY

## Abstract

**Objective:**

To investigate that whether an association between marital status and the female breast cancer risk exists.

**Methods:**

The MEDLINE, EMBASE and PsycINFO databases were searched from their inception to July 2019. The Newcastle-Ottawa Scale was used to rate the methodological quality of included studies. Study data were pooled using random-effects meta-analyses to compare the breast cancer risk between unmarried, widowed, divorced or lifelong single women and married women. This study is registered with PROSPERO (number CRD42018112368).

**Results:**

Forty-nine publications were included in the meta-analysis. Compared with married women, unmarried and lifelong single women had an elevated risk of breast cancer, and the pooled ORs of case-control studies were 1.20 (95% CI: 1.07 to 1.35) and 1.24 (95% CI: 1.05 to 1.45), respectively. In the subgroup analyses under these two comparisons, hospital-based estimates and multivariate-adjusted estimates demonstrated a strong association, while population-based estimates and age-adjusted estimates produced nonsignificant results. The pooled OR of cohort studies examining the effect of being a lifelong single woman was 1.10 (95% CI: 1.04 to 1.16). Heterogeneity was moderate to substantial across case-control studies (I^2^: 46% to 82%), which may be partially explained by differences in geographic regions, publication years and control types. Possible publication bias was indicated by the funnel plot and Egger’s test (P = 0.03).

**Conclusions:**

Marital status may correlate with the risk of developing female breast cancer. However, suboptimal selection of controls, insufficient exploration of confounding effects, inadequate ascertainment of marital status, and possible publication bias may have limited the quality of the available evidence. Overall, conclusions that marital status is an independent risk factor for breast cancer could not be drawn, and further prospective rigorous cohort studies are warranted.

## Introduction

Breast cancer is the most frequently diagnosed cancer and the leading cause of cancer death among women worldwide [1]. Approximately 52.7% of premenopausal breast cancer cases and 54.7% of postmenopausal breast cancer cases can be attributed to physiological, behavioral or genetic risk factors [2]. Feasible changes in risk behaviors, such as alcohol consumption, physical activity and obesity, can contribute to an important reduction in mammary carcinoma risk [3,4]. Under these circumstances, further progress may be achieved by identifying new, modifiable lifestyle-related risk factors.

One factor that may be associated with breast cancer is marital status. Married individuals typically enjoy a higher socioeconomic status than unmarried individuals, which may translate into better access to healthcare [5]. Marriage could also promote healthier lifestyle behaviors, such as regular screenings, healthy diet, and exercise, all of which may be mediating factors preventing breast cancer [6]. The situation is different for the unmarried people. For instance, being widowed or divorced often leaves individuals with a period of intense suffering and induces a series of unhealthy coping approaches that can account for the development of breast cancer [7]. Additionally, lifelong single women tend to have no experience with childbirth or breastfeeding, while parity, age at first full-term birth and the duration of breastfeeding have been proven to have a substantial influence on the incidence of breast cancer [8–10].

Some epidemiological studies have detected higher rates of breast cancer in unmarried people than married people [11,12], while some studies argue that marital status has no influence on this malignant disease [13,14]. Despite the disparity in previous results, no systematic research has been carried out. Thus, we conducted this systematic review and meta-analysis of observational studies to obtain valid knowledge regarding the associations between marital status and the risk of breast cancer in women.

## Materials and methods

We conducted a systematic review and meta-analysis in accordance with the MOOSE [15] (S1 Table) and PRISMA guidelines (S2 Table) [16]. The protocol was prospectively registered in the PROSPERO register of systematic reviews (CRD42018112368).

### Search strategy

The MEDLINE, EMBASE and PsycINFO databases were searched for electronic journals. The search duration was from the inception of the databases to July 2019. The search strategy included terms related to marital status and breast cancer combined with SIGN filters for observational studies (http://www.sign.ac.uk/search-filters.html). We confined our search to papers published in English. The reference lists of all eligible articles were also checked to identify additional studies. The search strategy is shown in S3 Table.

### Inclusion/exclusion criteria

The studies that fulfilled the following criteria were included:

1. studies employing observational research designs, including cohort, case-control, and cross-sectional designs, using appropriate controls,
2. studies involving at least two groups of married people and people with another status, including divorced, widowed, and lifelong single, or an aggregated category of all unmarried individuals,
3. studies presenting the results of analyses adjusted at least for age or studies where the control subjects were matched to cases by age; we contacted the authors of studies reporting unadjusted results and included new adjusted data if provided, and
4. studies published in English.

The studies that fulfilled the following criteria were excluded:

1. studies that did not establish a control group comprising participants without breast cancer, and
2. studies with control subjects matched to cases by marital status.

When two articles reported data from the same study, to avoid duplication, we only used the analysis with the higher methodological quality.

### Data extraction

Two reviewers (MLL and ZZL) independently assessed the titles, abstracts and keywords of each record retrieved. The full texts of all potentially relevant articles were investigated. Disagreements were resolved by discussion between the two reviewers and, if necessary, a third reviewer (SQZ).

The data were independently extracted from the included trials by two reviewers (MLL and NZ) and entered into a structured characteristics table. The extracted data included the following: the name of the first author, publication year, study design, features of the study population, strategies used to confirm breast cancer, assessment of marital status, research findings and other required information. We resolved any differences in opinion through consultation with a third person (MH).

### Quality assessment

We rated the methodological quality of the included studies using an adapted version of the Newcastle-Ottawa Scale (NOS) [17]. The NOS consists of eight items focusing on the following three domains: selection of study groups, ascertainment of exposure and outcome, and comparability of groups. The ratings are based on a star system with a maximum rating of nine. The assessments were performed by two authors (MLL and JTL), and any disagreements were resolved by discussion with a third author (SQZ).

### Statistical analyses

We provided a narrative synthesis of the findings from the included studies and pooled the results if the studies adopted the same methods to categorize marital status. Married women were used as the reference category. In all analyses, we generated inverse-variance weighted random-effects models with the DerSimonian-Laird estimator to account for the high expected heterogeneity across studies resulting from differences in the samples, measures, and designs. Subgroup analyses by control type (population-based studies versus hospital-based studies) and by adjustment level (multivariate-adjusted estimates versus age-adjusted estimates) were performed to determine the association between marital status and the risk of breast cancer.

The odds ratios (ORs) were used to measure the effect. If a study where the control subjects were matched to cases by age did not report the ORs, we calculated the ORs using a 2 × 2 cross tabulation. If a study did not use married people as the reference group, we inverted the ORs based on the method proposed by Greenland and Longnecker [18] with Microsoft Excel software developed by Hamling et al [19]. For studies that provided estimates of the relative risk based on different multivariate models, we prioritized the results from the model with the largest number of covariates.

Heterogeneity was tested using the Cochran Q statistic and quantified with the I-squared (I^2^) statistic, which describes the variation in the effect size attributable to the heterogeneity across studies. The confidence intervals for I^2^ were also calculated using the formula proposed by Higgins [20]. Low, moderate, and high degrees of heterogeneity were indicated by I^2^ values of <25%, 25–75%, and >75%, respectively. The potential sources of heterogeneity were explored if high heterogeneity was detected using a random-effects-weighted meta-regression based on the following variables: (1) adjustment level: multivariate-adjusted estimates versus age-adjusted estimates; (2) control type: population-based studies versus hospital-based studies; (3) geographic region: Asian versus non-Asian populations; and (4) publication year (continuous). Funnel plots and Egger’s test results were generated to detect publication bias for the comparison with the largest number of studies. Review Manager V.5.3 (Cochrane Collaboration) and Stata SE.15 software were used for the statistical analyses.

## Results

### Study selection

**Fig 1:**
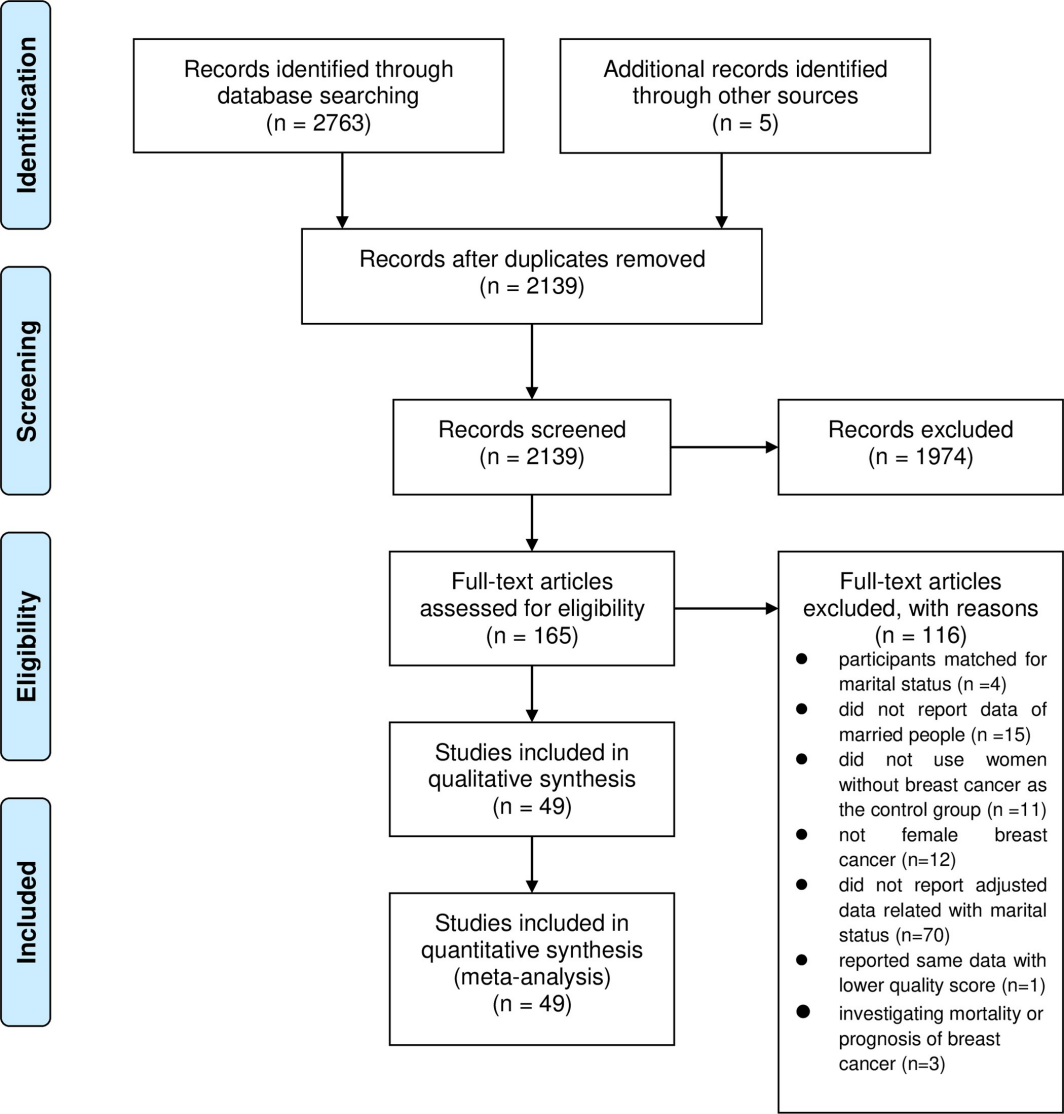
Flow diagram.

We identified 2763 articles using the search strategies. By reviewing the articles’ reference lists, we found five additional articles. After removing the duplicates and reviewing the titles and abstracts, we retrieved 165 full-text articles. Of these articles, 49 studies [11–14,21–65] fulfilled the inclusion criteria and were included in the meta-analysis (Fig 1).

### Description of studies

Table 1 describes the key study characteristics. We included two cohort studies [11,13] and 47 case-control studies [12,14,21–65]. The 49 studies included in our analyses involved 1,723,739 women, including 59,992 breast cancer cases. The studies were published between 1978 and 2018 and included participants from Europe, the United States, Australia, Africa and Asia. Overall, 19 studies [14,21,25,29,33,37,38,40–43,46,48,51,52,56,57,60,63] only provided age-adjusted estimates, while 30 studies [11–13,22–24,26–28,30–32,34–36,39,44,45,47,49,50,53–55,58,59,61,62,64,65] controlled for multiple confounding factors, such as demographic characteristics, reproductive factors, behavior and lifestyle factors and several psychological variables (full details are displayed in S4 Table). 20 studies (i.e. population-based studies) [11–14,21,26,29,33,34,36,38,40,41,46,49,51,54,58,62,64] recruited controls from the same community as cancer cases, while 29 studies (i.e. hospital-based studies) [22–25,27,28,30–32,35,37,39,42–45,47,48,50,52,53,55–57,59–61,62,65] selected controls from hospitals or other health care centers. Married people accounted for between 14.3% and 97.9% of the sample (widowed = 2.9% to 31.1%, divorced = 0.6% to 20.2%, and lifelong single = 0.7% to 53.9%).

**Table 1:** Characteristics of the included studies.

Study	Sample size		Region	Resource		Matched/adjusted for	Marital status (%)				NOS score
	Total	Cases		Case	Control		Married	Widowed	Divorced	Lifelong single	
**Cohort studies**											
Carlsen et al. [11]	1,590,000	25,855	Europe	population	population	multiple variables	N/A	N/A	N/A	N/A	7
Melchior et al. [13]	5493	120	Europe	employees	employees	multiple variables	N/A	N/A	N/A	N/A	7
**Case-control studies**											
Adami et al. [21]	358	179	Europe	population	population	age	60.3	24.9	4.2	10.6	6
Balekouzou et al. [22]	519	174	Africa	hospital	hospital	multiple variables	14.3	85.7 (w/d/lls)			6
Bano et al. [23]	1246	1238	Asia	hospital	hospital	multiple variables	N/A	N/A	N/A	N/A	4
Budiningsih et al. [24]	900	300	Asia	hospital	hospital	multiple variables	N/A	N/A	N/A	N/A	5
Cho et al. [25]	705	358	Asia	hospital	screening	age	81.8	13.3 (w/d)		4.8	5
Dey et al. [26]	2108	900	Asia	hospital	companion	multiple variables	83.2	N/A	14.9	1.9	5
Dianatinasab et al. [27]	1052	526	Asia	hospital	hospital	multiple variables	92.8	N/A	N/A	7.2	7
Ebrahimi et al. [28]	535	286	Asia	hospital	hospital	multiple variables	77.4	16.8 (w/d)		5.8	4
Ewertz et al. [14]	3520	1782	Europe	population	population	age	73.9	10.6	8.8	6.6	6
Faheem et al. [29]	300	150	Asia	hospital	population	age	92.3	7.7 (w/d/lls)			3
Forsen [30]	174	87	Europe	hospital	hospital	multiple variables	N/A	N/A	N/A	N/A	4
Gajalakshmi and Shanta [31]	1062	293	Asia	hospital	hospital	multiple variables	96.5	3.5 (w/d/lls)			4
Ghiasvand et al. [32]	1042	521	Asia	hospital	hospital	multiple variables	84.0	6.9 (w/d)		9.1	5
Gilani and Kamal [33]	1480	498	Asia	hospital	population	age	96.6	3.4 (w/d/lls)			6
Hadjisavvas et al. [34]	2282	1109	Europe	population	population	multiple variables	84.8	10.8 (w/d)		4.3	7
Jafari-Mehdiabad et al. [35]	285	98	Asia	hospital	hospital	multiple variables	84.2	11.9	1.8	2.1	5
Justenhoven et al. [36]	1997	1021	Europe	population	population	multiple variables	68.0	26.7 (w/d)		5.3	5
Khalis et al. [37]	474	237	Africa	hospital	hospital	age	68.8	12.2	8.2	10.8	6
Khan et al. [38]	196	100	Asia	hospital	population	age	84.2	15.8 (w/d/lls)			4
Kvikstad et al. [12]	45,685	4491	Europe	population	population	multiple variables	83.6	2.9	13.4	N/A	7
Laing et al. [39]	992	503	USA	hospital	hospital	multiple variables	38.3	40.3 (w/d)		21.4	5
Li et al. [40]	1982	975	USA	population	population	age	53.1	31.1	12.4	3.4	4
Lotfi and Shobairi [41]	159	80	Asia	hospital	population	age	88.1	11.9 (w/d/lls)			5
Mahouri et al. [42]	672	168	Asia	hospital	hospital	age	82.3	13.8 (w/d)		3.9	4
Marzouk et al. [43]	351	198	Europe	hospital	hospital	age	74.6	18.8	5.1	1.4	5
Mohite et al. [44]	434	217	Asia	hospital	hospital	multiple variables	85.0	10.6	2.3	2.1	4
Morales et al. [45]	1084	465	USA	hospital	hospital	multiple variables	61.1	6.5	20.2	12.3	6
Motie et al. [46]	254	134	Asia	population	population	age	97.2	2.8 (w/d/lls)			4
Oran et al. [47]	1244	622	Europe	hospital	hospital	multiple variables	81.3	N/A	N/A	6.0	5
Pakseresht et al. [48]	332	115	Asia	hospital	hospital	age	80.1	19.9 (w/d/lls)			5
Parameshwari et al. [49]	100	20	Asia	population	population	multiple variables	95.0	5.0 (w/d/lls)			4
Peled et al. [50]	622	255	Europe	hospital	hospital	multiple variables	72.7	20.9 (w/d/lls)			4
Pimhanam et al. [51]	888	444	Asia	hospital	population	age	67.5	10.0 (w/d)		22.5	4
Price et al. [52]	504	239	Australia	suspicion	suspicion	age	68.5	12.5	12.1	6.9	4
Rao et al. [53]	1400	689	Asia	hospital	hospital	multiple variables	76.4	20.6	0.6	2.4	4
Rookus and van Leeuwen [54]	1836	918	Europe	population	population	multiple variables	88.1	11.9 (w/d/lls)			5
Shamsi et al. [55]	883	297	Asia	hospital	hospital	multiple variables	N/A	N/A	N/A	N/A	6
Shaukat et al. [56]	94	42	Asia	hospital	hospital	age	97.9	2.1 (w/d/lls)			4
Sufian et al. [57]	216	108	Asia	hospital	hospital	age	93.5	6.5 (w/d/lls)			4
Tazhibi et al. [58]	257	216	Asia	relatives	relatives	multiple variables	94.2	5.8 (w/d/lls)			5
Tehranian et al. [59]	624	312	Asia	hospital	hospital	multiple variables	83.8	N/A	1.6	14.6	4
Thompson et al. [60]	1076	541	USA	hospital	screening	age	60.9	39.1 (w/d/lls)			5
Wakai et al. [61]	678	226	Asia	hospital	hospital	multiple variables	78.3	17.3	3.7	0.7	4
White et al. [62]	1607	747	USA	population	population	multiple variables	73.0	15.8 (w/d)		11.2	7
Yan et al. [63]	1042	521	Asia	hospital	hospital	age	93.5	6.5 (w/d/lls)			6
Eaker et al. [64]	28566	4761	Europe	population	population	multiple variables	62.9	2.9	17.8	16.8	8
Randi et al. [65]	14429	5856	Europe	hospital	hospital	multiple variables	N/A	N/A	N/A	N/A	7

N/A: not applicable.

### Methodological quality

In our review, the diagnosis of breast cancer was considered definitive if confirmed by pathological records. Guided by this criterion, all studies were deemed qualified and rated one star, except for three studies [40,49,54]. For the item regarding the ascertainment of exposure, studies using a secure record or structured questionnaire with details regarding the timing of potential changes in marital status were rated one star. Almost all studies failed to meet this standard, except for three studies that used regularly updated marriage registry records [11,14,64]. The studies were rated two stars in the domain of comparability if they adjusted for at least three of the following known breast cancer risk factors: family history of breast cancer, parity, usage of hormone replacement therapy, usage of oral contraceptives, age at first and final birth, number of live births/abortions/miscarriages, birth interval, history of breastfeeding, lifelong menstrual pattern, menopausal status, age at menarche/menopause, education level, body mass index (BMI), alcohol intake and smoking. All studies controlled for age, but only 12 studies [13,22,27,32,34,36,39,45,47,55,58,65] were considered to have adequate comparability between the cases and controls.

Among the cohort studies, the studies selecting participants from a population or community with an initial response rate over 70% were considered adequately representative. Thus, one study [11] comprising all eligible residents in a country was rated one star, while the other study [13] in which only 45% accepted and completed a baseline survey received no stars. Regarding the second selection item, both studies obtained the non exposed cohort from the same source as the exposed cohort. In addition, both studies demonstrated that the outcome of interest was not present at the start of the study. Concerning follow-up, one study [11] followed up for less than 10 years with a dropout rate of 5.6%, while the other study [13] followed up for an average of 10.6 years with a dropout rate of 1%.

Among the case-control studies, 19 studies [12,21,24,27,30,32,33,35,37,41,43,44,46,48,54,55,62,64,65] were rated one star for the item of representativeness of the cases since they selected consecutive eligible cases in a defined area over a defined period. However, selection bias was still possible as only 12 studies recruited controls from the same community as the cases, while the remaining studies either used a hospital-based design [22–24,27,28,30–32,35,39,42–45,47,48,50,52,53,55–57,59,60,63,65] or provided no description of the source [25,26,29,37,38,46,51,58,60]. Eight studies [14,29,30,36,40,41,44,46] did not receive a star in the domain of control definition since these studies did not clearly demonstrate that the controls had no history of breast cancer. 18 studies reported a nonresponse rate with details, including 14 studies [12,14,22,25–27,34,37,40,45,60,62,63,65] with the same rate (difference less than 10%) between the cases and controls, which were rated one star.

The mean methodological quality score of the cohort studies and case-control studies was 7/9 and 5/9, respectively. Overall, both designs failed to demonstrate a satisfying performance in the domain of ascertainment of exposure and comparability of participants. The case-control studies also scored poorly on the item related to the representativeness of the cases and selection of controls. The full details of the methodological assessment are shown in S5 Table.

### Effect estimates

Table 2 displays a summary of the effect estimates of the association between marital status and risk of breast cancer. Since the method used to categorize marital status varied across studies, we performed meta-analyses using the following comparisons:

1. unmarried (an aggregated category including widowed, divorced and lifelong single) versus married people (n = 41);
2. widowed versus married people (n = 14);
3. divorced versus married people (n = 16); and
4. lifelong single (i.e., never married) versus married people (n = 27).

**Table 2:** Summary of effect estimates.

Subgroups		No. of studies	Pooled estimates	P-value	heterogeneity	
					I^2^	P-value
**Unmarried**						
Cohort		0	/	/	/	/
case-control	total	41	1.20 [1.07, 1.35]	0.002	82% [77%, 87%]	< 0.00001
	population-based	17	1.13 [0.99, 1.29]	0.07	80% [68%, 87%]	< 0.00001
	hospital-based	24	1.23 [1.01, 1.50]	0.04	81% [72%, 87%]	< 0.00001
	subgroup differences			0.49		
**Widowed**						
cohort	(population-based)	1	0.98 [0.93, 1.03]	0.44	/	/
case-control	total	13	0.96 [0.86, 1.08]	0.51	46% [0%, 72%]	0.03
	population-based	5	0.99 [0.87, 1.13]	0.89	44% [0%, 80%]	0.13
	hospital-based	8	0.94 [0.75, 1.18]	0.60	47% [0%, 77%]	0.06
	subgroup differences			0.70		
**Divorced**						
cohort	(population-based)	1	1.04 [0.99, 1.09]	0.08	/	/
case-control	total	15	1.16 [0.96, 1.39]	0.12	80% [67%, 87%]	< 0.00001
	population-based	6	1.03 [0.84, 1.26]	0.79	85% [70%, 93%]	< 0.00001
	hospital-based	9	1.66 [1.02, 2.70]	0.04	77% [55%, 88%]	< 0.0001
	subgroup differences			0.08		
**Lifelong single**						
cohort	(population-based)	2	1.10 [1.04, 1.16]	0.0004	0% [0%, 23%]	0.33
case-control	total	25	1.24 [1.05, 1.45]	0.01	69% [54%, 80%]	< 0.00001
	population-based	9	1.00 [0.93, 1.08]	0.97	0% [0%, 99%]	0.50
	hospital-based	16	1.51 [1.07, 2.13]	0.02	79% [66%, 87%]	< 0.00001
	subgroup differences			0.02		
**Unmarried**						
Cohort		0	/	/	/	/
case-control	total	41	1.20 [1.07, 1.35]	0.002	82% [77%, 87%]	< 0.00001
	multi-adjusted	22	1.23 [1.05, 1.45]	0.01	88% [83%, 91%]	< 0.00001
	age-adjusted	19	1.16 [0.99, 1.37]	0.07	65% [43%, 78%]	< 0.0001
	subgroup differences			0.61		
**Widowed**						
cohort	(multi-adjusted)	1	0.98 [0.93, 1.03]	0.44	/	/
case-control	total	13	0.96 [0.86, 1.08]	0.51	46% [0%, 72%]	0.03
	multi-adjusted	7	1.02 [0.84, 1.23]	0.85	67% [28%, 85%]	0.005
	age-adjusted	6	0.89 [0.79, 1.02]	0.09	0% [0%, 64%]	0.80
	subgroup differences			0.27		
**Divorced**						
cohort	(multi-adjusted)	1	1.04 [0.99, 1.09]	0.08	/	/
case-control	total	15	1.16 [0.96, 1.39]	0.12	80% [67%, 87%]	< 0.00001
	Multi-adjusted	9	1.25 [0.97, 1.61]	0.08	87% [78%, 93%]	< 0.00001
	age-adjusted	6	1.02 [0.83, 1.27]	0.82	20% [0%, 64%]	0.28
	subgroup differences			0.23		
**Lifelong single**						
cohort	(multi-adjusted)	2	1.10 [1.04, 1.16]	0.0004	0% [0%, 23%]	0.33
case-control	total	25	1.24 [1.05, 1.45]	0.01	69% [54%, 80%]	< 0.00001
	multi-adjusted	16	1.28 [1.04, 1.58]	0.02	76% [62%, 85%]	< 0.00001
	age-adjusted	9	1.17 [0.91, 1.49]	0.22	39% [0%, 72%]	0.11
	subgroup differences			0.56		

#### Unmarried versus married women

**Fig 2:**
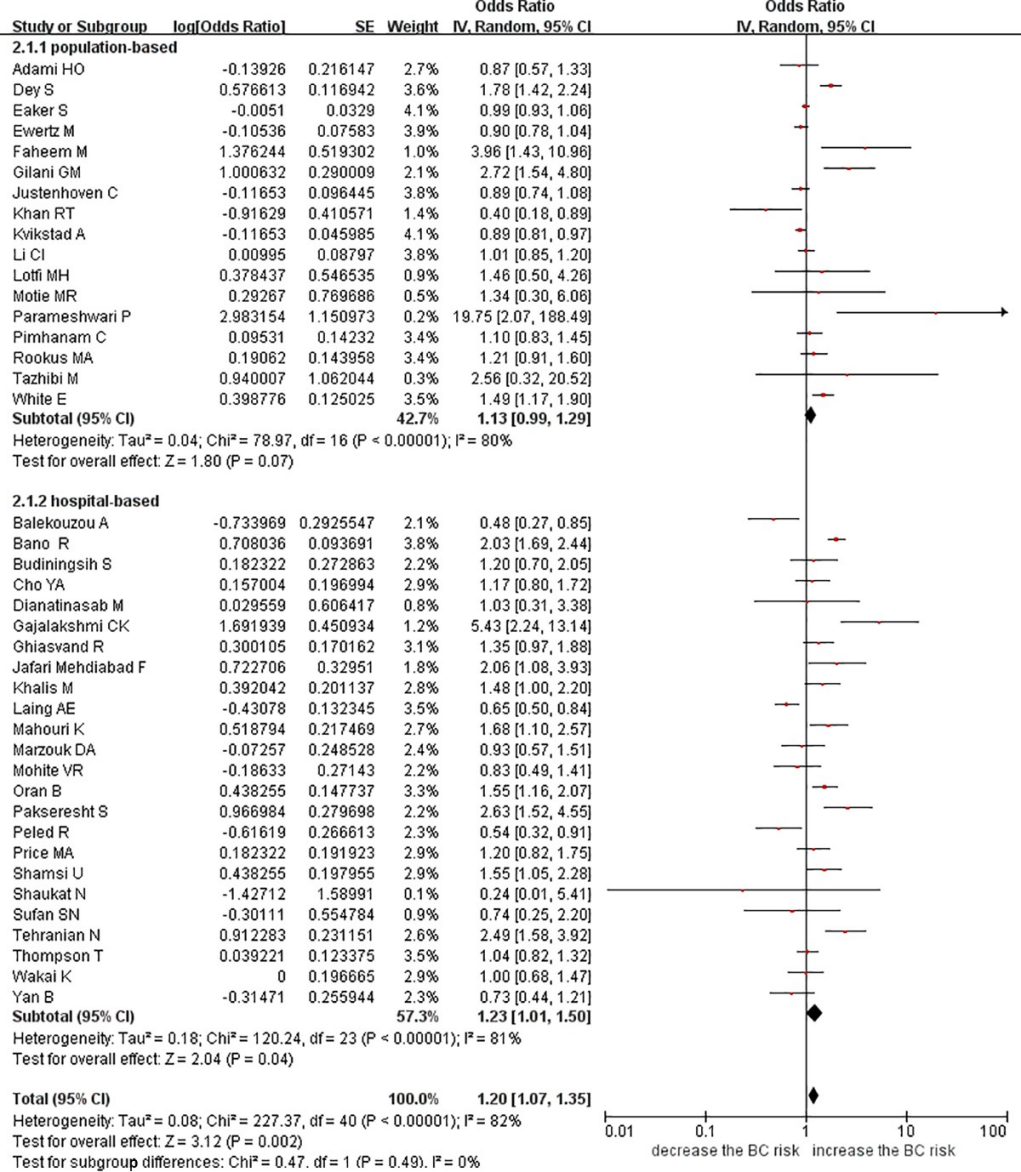
Forest plot of breast cancer risk among unmarried women versus married women stratified by control type.

No eligible cohort study was included in this comparison. In the 41 case-control studies [12,14,21–27,29,31–33,35–44,46–52,54–64], a 20% risk increase (OR = 1.20; 95% confidence interval (95% CI): 1.07 to 1.35) for breast cancer was detected in the unmarried women versus the married women, with considerable heterogeneity (I^2^ = 82%; 95% CI: 77% to 87%). The meta-regression identified geographic region as a potential source of heterogeneity (P = 0.004) (S2 File). When we stratified our analysis by control type, we found a persistent positive association among the hospital-based studies (OR = 1.23, 95% CI: 1.01 to 1.50; I^2^ = 81%, 95% CI: 72% to 87%; n = 24), but no significant association was observed among the population-based studies (OR = 1.13, 95% CI: 0.99 to 1.29; I^2^ = 80%, 95% CI: 68% to 87%; n = 17). However, the subgroup difference was not significant (P = 0.49) (Fig 2). According to another subgroup analysis, the studies that adjusted for multiple variables resulted in an OR of 1.23 (95% CI: 1.05 to 1.45; I^2^ = 88%, 95% CI: 83% to 91%), whereas the studies that adjusted for age produced a nonsignificant OR of 1.16 (95% CI: 0.99 to 1.37; I^2^ = 65%, 95%CI: 43% to 78%). However, a subgroup difference was not detected (P = 0.61) (S3 File).

#### Widowed versus married women

According to the results of the cohort study [11], the breast cancer risk in widowed people did not differ from that in married people (relative risk (RR) = 0.98; 95% CI: 0.93 to 1.03). The analysis of the 13 case-control studies [12,14,21,35,37,40,43–45,52,61,64,65] yielded a similar combined OR of 0.96 (95% CI: 0.86 to 1.08) with moderate between-study heterogeneity (I^2^ = 46%, 95% CI: 0% to 72%). Subgroup analyses did not identify any statistically significant association between being widowed and the breast cancer risk. The population-based studies produced an OR of 0.99 (95% CI: 0.87 to 1.13; I^2^ = 44%, 95% CI: 0% to 80%; n = 5) while the hospital-based studies produced an OR of 0.94 (95% CI: 0.75 to 1.18; I^2^ = 47%, 95% CI: 0% to 77%; n = 8). The studies with maximal adjustment produced an OR of 1.02 (95% CI: 0.84 to 1.23; I^2^ = 67%, 95% CI: 28% to 85%; n = 7) while the studies that adjusted for age produced an OR of 0.89 (95% CI: 0.79 to 1.02; I^2^ = 0%, 95% CI: 0% to 64%; n = 6). A significant subgroup difference was not observed in both stratified analyses (P = 0.70; P = 0.27) (S3 File).

#### Divorced versus married women

One cohort study [11] investigated the association between divorced status and the breast cancer risk and provided an RR of 1.04 (95% CI: 0.99 to 1.09), while 15 case-control studies [12,14,21,26,35,37,40,43–45,52,59,61,64,65] yielded a nonsignificant pooled OR of 1.16 (95% CI: 0.96 to 1.39; I^2^ = 80%, 95% CI: 67% to 87%). The meta-regression showed that geographic region (P = 0.017) and publication year (P = 0.024) were significantly associated with the effect estimates and may have been the causes of heterogeneity (S2 File). The hospital-based subgroup analysis produced an OR of 1.66 (95% CI: 1.02 to 2.70; I^2^ = 77%, 95% CI: 55% to 88%; n = 9), while the population-based analysis produced a nonsignificant OR of 1.03 (95% CI: 0.84 to 1.26; I^2^ = 85%, 95% CI: 70% to 93%; n = 6). The multivariate-adjusted subgroup analysis resulted in an OR of 1.25 (95% CI: 0.97 to 1.61; I^2^ = 87%, 95% CI: 78% to 93%; n = 9), whereas the age-adjusted analysis produced a nonsignificant OR of 1.02 (95% CI: 0.83 to 1.27; I^2^ = 20%, 95% CI: 0% to 64%; n = 6). A significant subgroup difference was not observed in both stratified analyses (P = 0.08; P = 0.23) (S3 File).

#### Lifelong single versus married women

Two cohort studies [11,13] investigated the association between lifelong single status and the breast cancer risk and provided an OR of 1.10 (95% CI: 1.04 to 1.16; I^2^ = 0%, 95% CI: 0% to 23%), while 25 case-control studies [14,21,25,26,28,30,32,34–37,39,40,42–45,47,51,52,59,61,62,64,65] yielded an OR of 1.24 (95% CI: 1.05 to 1.45; I^2^ = 69%, 95% CI: 54% to 80%). The subgroup analysis according to control type detected a significant subgroup difference (P = 0.02), but the subgroup analysis according to adjustment level failed to detect any difference (P = 0.56). The hospital-based studies produced a combined OR of 1.51 (95% CI: 1.07 to 2.13; I^2^ = 79%, 95% CI: 66% to 87%; n = 16), while the population-based studies yielded a nonsignificant OR of 1.00 (95% CI: 0.93 to 1.08; I^2^ = 0%, 95% CI: 0% to 99%; n = 9). The multivariate-adjusted estimates increased the overall OR to 1.28 (95% CI: 1.04 to 1.58; I^2^ = 76%, 95% CI: 62% to 85%; n = 16), while the age-adjusted estimates produced a nonsignificant combined OR of 1.17 (95% CI: 0.91 to 1.49; I^2^ = 39%, 95% CI: 0% to 72%; n = 9) (S3 File).

#### Publication bias

**Fig 3:**
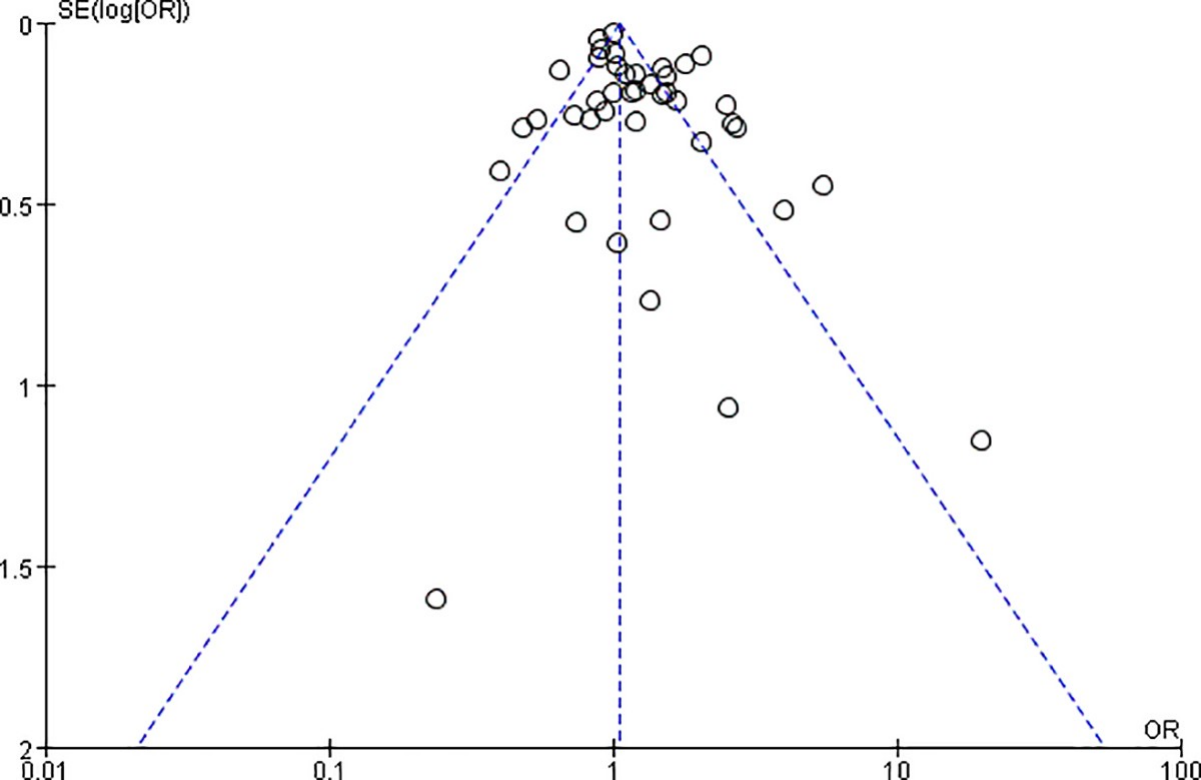
Funnel plot of the effect of being unmarried on the risk of breast cancer.

There was evidence of significant funnel plot asymmetry (Egger’s test: P = 0.03), suggesting publication bias among the studies that investigated the breast cancer risk in unmarried women (n = 41) (Fig 3).

## Discussion

### Main findings

Our study summarized all accessible published evidence and demonstrated that unmarried women, especially lifelong single women, had a higher risk of developing breast cancer than married women. Even though positive results were obtained, we could not draw the conclusion that marital status is an independent factor associated with breast cancer.

As indicated by the subgroup analysis where only hospital-based studies showed positive associations between marital status and the breast cancer risk, control type may affect the effect estimates. According to another subgroup analysis, the multivariate-adjusted estimates demonstrated a significant association in several comparisons, but the age-adjusted estimates revealed such associations in no comparisons. We speculate that these results might be attributed to interactions among the independent variables analyzed in the multivariable-adjusted models. Unfortunately, we could not investigate whether there are interactions since none of the original studies provided enough details.

Heterogeneity was moderate to substantial across the included case-control studies, which may be partly explained by geographic region, control type and publication year according to the subgroup analyses and meta-regression analyses. The results were consistent with those reported in previous studies. For instance, individuals of different races may experience different relationships between marriage and health [66], and the impact of marital status on the cancer incidence may also vary across cultures and change over time [67]. Moreover, the proportion of suspicious cases and the distribution of marital status were different between hospital-based studies and population-based studies, which supported the role of control type in the generation of between-study heterogeneity. Although several other factors, such as age and cancer subtypes, should also be considered, further analyses were not employed due to the limited data in the original studies.

We also identified possible publication bias, suggesting that some studies that failed to show a significant association between unmarried status and the breast cancer risk may have not been published.

### Strengths and limitations

Thus far, no formal attempts have been made to systematically review data regarding the potential relationship between marital status and the risk of breast cancer. Therefore, the data included in our analysis represent the only available evidence for the enrichment of clinical screening and prevention decisions. However, considerable caution is warranted when interpreting these results due to the limitations of this review.

First, most case-control studies used a hospital-based design, resulting in controls that often fail to reflect the distribution of key characteristics of the population from which the cases were drawn. Another concern is that retrospective studies are believed to be subject to recall bias caused by memory distortion. However, major life events, including divorce, bereavement and getting married [68], show minimal change in recall over time and are, therefore, associated with greater reporting reliability [69]. Moreover, few included studies have recorded details of the changes in and duration of the marital status; thus, whether the current status or previous status produced the effect observed in the studies could not be determined in this review. Consequently, our review could not confirm the temporal relationship between the recorded marital status and the initiation of breast cancer.

### Implications for practice and further research

Prospective cohort studies with sufficient details regarding the marital status and adequate adjustment for confounding factors should be carried out to explore the dose-response effect of different marital status categories and provide support for a causal relationship between marital status and breast cancer incidence. The moderating and mediating mechanisms of this relationship deserve substantial consideration to develop tailored interventions and strategies to detect breast cancer at an early stage.

## Conclusions

This review detects a higher rate of breast cancer in unmarried women, especially lifelong single women, than married women. However, the quality of the available data is limited by possible publication bias and several methodological drawbacks, including suboptimal selection of controls, insufficient exploration of confounding effects, and inadequate ascertainment of marital status. Overall, conclusions that marital status is an independent risk factor for breast cancer could not be drawn, and fully adjusted prospective cohort studies with sufficient details regarding marital status should be conducted in the future.

## Supporting information

S1 Table MOOSE checklist. (DOC)

S2 Table PRISMA checklist. (DOC)

S3 Table Search strategy. (DOCX)

S4 Table Confounding factors involved in the multivariate-adjusted studies. (DOCX)

S5 Table Quality assessment of the included studies. (DOC)

S1 File Protocol. (PDF)

S2 File Results of the meta-regression analyses. (DOCX)

S3 File Forest plots. (DOCX)
