# Supplementary material for: Does marital status correlate with the female breast cancer risk? A systematic review and meta-analysis of observational studies
Source: PLoS One. 2020 Mar 5;15(3):e0229899. doi: 10.1371/journal.pone.0229899 (PMC7058335; doi:10.1371/journal.pone.0229899)
Supplement: S1 Table — (DOC) [file pone.0229899.s001.doc]

**S1 Table. MOOSE Checklist.**

| **Item No.** | **Recommendation** | **Reported on page No.** |
| --- | --- | --- |
| **Reporting of background should include** | | |
| 1 | Problem definition | 5 |
| 2 | Hypothesis statement | 5 |
| 3 | Description of study outcomes | 5 |
| 4 | Type of exposure or intervention used | 5 |
| 5 | Type of study designs used | 5 |
| 6 | Study population | 5 |
| **Reporting of search strategy should include** | | |
| 7 | Qualifications of searchers (e.g., librarians and investigators) | 6 |
| 8 | Search strategy, including time period included in the synthesis and keywords | 6 |
|  |  | S3 Table |
| 9 | Effort to include all available studies, including contact with authors | 7 |
| 10 | Databases and registries searched | 6 |
| 11 | Search software used, name and version, including special features used (e.g., explosion) | 6 |
| 12 | Use of hand searching (e.g., reference lists of obtained articles) | 6 |
| 13 | List of citations located and those excluded, including justifications | Fig 1 |
| 14 | Method of addressing articles published in languages other than English | 6 |
| 15 | Method of handling abstracts and unpublished studies | 6 |
| 16 | Description of any contact with authors | 7 |
| **Reporting of methods should include** | | |
| 17 | Description of relevance or appropriateness of studies assembled for assessing the hypothesis to be tested | 7 |
| 18 | Rationale for the selection and coding of data (e.g., sound clinical principles or convenience) | 6, 7 |
| 19 | Documentation of how data were classified and coded (e.g., multiple raters, blinding and interrater reliability) | 7, 8 |
| 20 | Assessment of confounding (e.g., comparability of cases and controls in studies where appropriate) | 8 |
| 21 | Assessment of study quality, including blinding of quality assessors; stratification or regression on possible predictors of study results | 8 |
| 22 | Assessment of heterogeneity | 9 |
| 23 | Description of statistical methods (e.g., complete description of fixed or random effects models, justification of whether the chosen models account for predictors of study results, dose-response models, or cumulative meta-analysis) in sufficient detail to be replicated | 8, 9 |
| 24 | Provision of appropriate tables and graphics | Table 1, 2 |
|  |  | Figs 1-3  S7,8 Files |
| **Reporting of results should include** | | |
| 25 | Graph summarizing individual study estimates and overall estimate | Table 2 |
|  |  | Figs 2  S8 File |
| 26 | Table giving descriptive information for each study included | Table 1 |
|  |  | S4 Table |
| 27 | Results of sensitivity testing (e.g., subgroup analysis) | 19-21  Figs 2  S7, 8 Files |
| 28 | Indication of statistical uncertainty of findings | 19-21 |
| **Reporting of discussion should include** | | |
| 29 | Quantitative assessment of bias (e.g., publication bias) | 21  Fig 3 |
| 30 | Justification for exclusion (e.g., exclusion of non-English language citations) | 21 |
| 31 | Assessment of quality of included studies | 15-17 |
|  |  | S5 Table |
| **Reporting of conclusions should include** | | |
| 32 | Consideration of alternative explanations for observed results | 22-24 |
| 33 | Generalization of the conclusions (i.e., appropriate for the data presented and within the domain of the literature review) | 22, 23 |
| 34 | Guidelines for future research | 25 |
| 35 | Disclosure of funding source | / |
