# Supplementary material for: Does marital status correlate with the female breast cancer risk? A systematic review and meta-analysis of observational studies
Source: PLoS One. 2020 Mar 5;15(3):e0229899. doi: 10.1371/journal.pone.0229899 (PMC7058335; doi:10.1371/journal.pone.0229899)
Supplement: S3 Table — (DOCX) [file pone.0229899.s003.docx]

**S2 Table. Search strategy.**

| **MEDLINE <1946 to July 15, 2019> (n=1245)** | |
| --- | --- |
| #1 | Epidemiologic Studies/ |
| #2 | exp Case-Control Studies/ |
| #3 | exp Cohort Studies/ |
| #4 | Cross-Sectional Studies/ |
| #5 | (Case control or (cohort adj (study or studies)) or Cohort analy$ or (Follow up adj (study or studies)) or (observational adj (study or studies)) or Longitudinal or Retrospective or Cross sectional).tw. |
| #6 | (#1 or #2 or #3 or #4 or #5) |
| #7 | exp Breast Neoplasms/ |
| #8 | (Breast adj6 (cancer* or neoplasm* or carcinoma* or adenocarcinoma* or tumor* or tumour* or malignanc*)).ab,ti. |
| #9 | (#7 or #8) |
| #10 | exp Marital Status/ |
| #11 | (marital status or civil status or social integration or marriage or married or spouse or partner or confidant$).ab,ti,tw. |
| #12 | (widow or widowed or divorce or divorced or separated or cohabit or unmarried).ab,ti,tw. |
| #13 | (#11 or #12) |
| #14 | (#6 and #9 and #13) |
| **Embase <1947 to July 15, 2019> (n=1406)** | |
| #1 | Clinical study/ |
| #2 | Case control study/ |
| #3 | Longitudinal study/ |
| #4 | Retrospective study/ |
| #5 | Prospective study/ |
| #6 | Cohort analysis/ |
| #7 | (#1 or #2 or #3 or #4 or #5 or #6) |
| #8 | Randomized controlled trials/ |
| #9 | (#7 not #8) |
| #10 | ((Case control adj (study or studies)) or (follow up adj (study or studies)) or (observational adj (study or studies)) or (epidemiologic$ adj (study or studies)) or (cross sectional adj (study or studies)).tw. |
| #11 | (#9 or #10) |
| #12 | exp Breast Neoplasms/ |
| #13 | (Breast adj6 (cancer* or neoplasm* or carcinoma* or adenocarcinoma* or tumor* or tumour* or malignanc*)).ab,ti. |
| #14 | (#11 or #12) |
| #15 | exp marriage/ |
| #16 | (marital status or civil status or social integration or marriage or married or spouse or partner or confidant$).ab,ti,tw. |
| #17 | (widow or widowed or divorce or divorced or separated or cohabit or unmarried).ab,ti,tw. |
| #18 | (#15 or #16 or #17) |
| #19 | (#11 and #14 and #18) |
| **PsycINFO <1806 to July Week 2 2019> (n=112)** | |
| #1 | Longitudinal studies/ |
| #2 | (cohort or prospective or longitudinal or retrospective).mp. |
| #3 | ((case* adj5 control*) or (case adj3 comparison*) or case-comparison or control group*).ab,id,ti. |
| #4 | (#1 or #2 or #3) |
| #5 | "literature review".md. |
| #6 | (#4 not #5) |
| #7 | exp Breast Neoplasms/ |
| #8 | (Breast adj6 (cancer* or neoplasm* or carcinoma* or adenocarcinoma* or tumor* or tumour* or malignanc*)).ab,ti |
| #9 | (#7 or #8) |
| #10 | exp marriage/ |
| #11 | exp Marital Status/ |
| #12 | (marital status or civil status or social integration or marriage or married or spouse or partner or confidant$).ab,ti,tw. |
| #13 | (widow or widowed or divorce or divorced or separated or cohabit or unmarried).ab,ti,tw.) |
| #14 | (#10 or #11 or #12 or #13) |
| #15 | (#6 and #9 and #14) |
