# Supplementary material for: Does marital status correlate with the female breast cancer risk? A systematic review and meta-analysis of observational studies
Source: PLoS One. 2020 Mar 5;15(3):e0229899. doi: 10.1371/journal.pone.0229899 (PMC7058335; doi:10.1371/journal.pone.0229899)
Supplement: S4 Table — (DOCX) [file pone.0229899.s004.docx]

**S4 Table. Confounding factors involved in the multivariate-adjusted studies.**

| **Study** | **Controlling for** |
| --- | --- |
| Carlsen 2008 | calendar period, age, Edu and disposable income |
| Melchior 2005 | age, marital status, tobacco and alcohol consumption, weight, diet, family history of cancer, and reproductive history |
| Balekouzou 2017 | age, occupation, economic status, Edu, residence, and parity |
| Bano 2016 | age, ethnicity and geographical location |
| Budiningsih 1999 | age, socioeconomic status and race |
| Dey 2009 | age and residence status |
| Dianatinasab 2017 | age, Edu, occupation, family history of BC, smoking, second hand smoking, OC usage, chest X-ray, hair coloring, physical exercise, BMI, birth weight, marital age, age at first delivery, parity, birth interval, breastfeeding, menarche age, menopause status, stressful life, sleep quality and regular bedtime. |
| Ebrahimi 2002 | age, family history of BC, and parity |
| Forsen 1991 | age, sex, language and number of registered children, premorbid anxiety, premorbid depression, 12-month SRRS，important emotional loss, Edu, and social class |
| Gajalakshmi 1991 | age, socioeconomic class and menopausal status |
| Ghiasvand 2010 | age, residence, occupation, Edu, age at menarche, age at first marriage/pregnancy, parity, menopause status, breast feeding, OC usage, family history of BC and BMI |
| Hadjisavvas 2010 | age, Edu, BMI, family history of BC, age at menarche, age at 1st pregnancy, breastfeeding, OC use, HRT use, exercise and smoking. |
| Jafari Mehdiabad 2017 | age and place of residence |
| Justenhoven 2010 | age, menopausal status, family history of BC, OC use, HRT use, BMI, and smoking |
| Kvikstad 1994 | age, age at first birth and parity for widowed women; further adjusted for place of residence for divorced women |
| Laing 1993 | age, age at diagnosis, age at menarche, parity, induced abortion, miscarriage, menopausal status, and family history |
| Mohite 2015 | age, religion and residence |
| Morales 2013 | age, BMI, family history of BC, menopause, number of children, alcohol use, smoking and vitamin use |
| Oran 2001 | age, menopausal status, age at menopause, age at menarche, BMI, OC use, family history of BC, history of BBDs |
| Parameshwari 2013 | age and geographic area |
| Peled 2008 | age, exposure to more than one life event, the feeling of happiness and optimism |
| Rao 1994 | age and place of residence. |
| Rookus 1994 | age and area |
| Shamsi 2013 | age, hospital, age at menarche, parity, age at 1st full-term pregnancy, miscarriage, breastfeeding, abortion, OC use and HRT use. |
| Tazhibi 2018 | vocation, age, menopause status, OC use and HRT use |
| Tehranian 2010 | age and ethnicity |
| Wakai 2000 | age and socioeconomic status |
| White 1994 | age and county of residence |
| Eaker 2011 | age and community |
| Randi 2004 | age, study center, area of residence, education, smoking habit, and alcohol intake |

BBD: benign breast disease; BC: breast cancer; BMI: body mass index; Edu: education; HRT: hormone replacement therapy;
