# Supplementary material for: Does marital status correlate with the female breast cancer risk? A systematic review and meta-analysis of observational studies
Source: PLoS One. 2020 Mar 5;15(3):e0229899. doi: 10.1371/journal.pone.0229899 (PMC7058335; doi:10.1371/journal.pone.0229899)
Supplement: S5 Table — (DOC) [file pone.0229899.s005.doc]

**S5 Table. Quality assessment. (A)cohort studies.**

| **Author** | **Selection** | | | | **Comparability** | | **Outcome** | | | **Overall quality** |
| --- | --- | --- | --- | --- | --- | --- | --- | --- | --- | --- |
|  | **Representative cohort** | **Control selection** | **Exposure assessment** | **Outcome exclusion** | **Control for age** | **Control adequately** | **Outcome assessment** | **FU length** | **FU availability** |  |
| Carlsen [11] | * | * | *a | * | * | / | *b | b | * | 7 |
| Melchior [13] | b | * | d | * | * | * | *b | * | * | 7 |

**Selection**

1) Representativeness of the exposed cohort

a) truly representative of the average women in the community with initial response rate over 70% *****

b) selected group of users (e.g. nurses, volunteers)

c) no description of the derivation of the cohort

2) Selection of the non exposed cohort

a) drawn from the same community as the exposed cohort *****

b) drawn from a different source

c) no description of the derivation of the non exposed cohort

3) Ascertainment of exposure

a) secure record (e.g. regularly-updated marriage registry records) ***** ****

b) structured questionnaire with details on timing of potential changes of marital status***** ****

c) interview not blinded to breast cancer status

d) written self-report or medical record only

e) no description

4) Demonstration that outcome of interest was not present at start of study

a) yes *****

b) no

**Comparability**

1) Comparability of cohorts on the basis of the design or analysis

a) study controls for age***** ****

b) study also controls for at least three of the following known breast cancer risk factors: family history of BC, parity, OC/HRT usage, age at first and last birth, number of live birth/abortion/miscarriages, birth interval, breastfeeding, lifelong menstrual pattern, menopausal status, age at menarche/menopause, Edu, BMI, alcohol intake, smoking ******

**Outcome**

1) Assessment of outcome

a) independent blind assessment***** ****

b) record linkage***** ****

c) self-report

d) no description

2) Was follow-up long enough for outcomes to occur

a) at least 10 years***** ****

b) no

3) Adequacy of follow up of cohorts

a) complete follow up - all subjects accounted for***** ****

b) less than < 30% lost to follow up*****

c) follow up rate < 70% and no description of those lost

d) no statement

**S5 Table. Quality assessment. (B)case-control studies.**

| **Author** | **Selection** | | | | **Comparability** | | **Exposure** | | | **Overall quality** |
| --- | --- | --- | --- | --- | --- | --- | --- | --- | --- | --- |
|  | **Case definition** | **Case representativeness** | **Control selection** | **Control definition** | **Control for age** | **Control adequately** | **Exposure assessment** | **Same method** | **Non-Response Rate** |  |
| Adami [21] | * | * | * | * | * | / | d | * | b | 6 |
| Balekouzou [22] | * | b | b | * | * | * | d | * | * | 6 |
| Bano [23] | * | b | b | * | * | / | d | * | b | 4 |
| Budiningsih [24] | * | * | b | * | * | / | d | * | b | 5 |
| Cho [25] | * | b | c | * | * | / | d | * | * | 5 |
| Dey [26] | * | b | c | * | * | / | d | * | * | 5 |
| Dianatinasab [27] | * | * | b | * | * | * | d | * | * | 7 |
| Ebrahimi [28] | * | b | b | * | * | / | d | * | b | 4 |
| Ewertz [14] | * | b | * | b | * | / | * | * | * | 6 |
| Faheem [29] | * | b | c | b | * | / | d | * | b | 3 |
| Forsen [30] | * | * | b | b | * | / | d | * | b | 4 |
| Gajalakshmi [31] | * | b | b | * | * | / | d | * | b | 4 |
| Ghiasvand [32] | * | * | b | * | * | * | d | * | b | 6 |
| Gilani [33] | * | * | * | * | * | / | d | * | b | 6 |
| Hadjisavvas [34] | * | b | * | * | * | * | d | * | * | 7 |
| Jafari-Mehdiabad [35] | * | * | b | * | * | / | d | * | b | 5 |
| Justenhoven [36] | * | b | * | b | * | * | d | * | c | 5 |
| Khalis [37] | * | * | c | * | * | / | d | * | * | 6 |
| Khan [38] | * | b | c | * | * | / | d | * | b | 4 |
| Kvikstad [12] | * | * | * | * | * | / | d | * | * | 7 |
| Laing [39] | * | b | b | * | * | * | d | * | b | 5 |
| Li [40] | b | b | * | b | * | / | d | * | * | 4 |
| Lotfi [41] | * | * | * | b | * | / | d | * | b | 5 |
| Mahouri [42] | * | b | b | * | * | / | d | * | b | 4 |
| Marzouk [43] | * | * | b | * | * | / | d | * | b | 5 |
| Mohite [44] | * | * | b | b | * | / | d | * | b | 4 |
| Morales [45] | * | b | b | * | * | * | d | * | * | 6 |
| Motie [46] | * | * | c | b | * | / | d | * | b | 4 |
| Oran [47] | * | b | b | * | * | * | d | * | b | 5 |
| Pakseresht [48] | * | * | b | * | * | / | d | * | b | 5 |
| Parameshwari [49] | b | b | * | * | * | / | d | * | b | 4 |
| Peled [50] | * | b | b | * | * | / | d | * | b | 4 |
| Pimhanam [51] | * | b | c | * | * | / | d | * | b | 4 |
| Price [52] | * | b | b | * | * | / | d | * | b | 4 |
| Rao [53] | * | b | b | * | * | / | d | * | b | 4 |
| Rookus [54] | b | * | * | * | * | / | d | * | c | 5 |
| Shamsi [55] | * | * | b | * | * | * | d | * | b | 6 |
| Shaukat [56] | * | b | b | * | * | / | d | * | b | 4 |
| Sufian [57] | * | b | b | * | * | / | d | * | b | 4 |
| Tazhibi [58] | * | b | c | * | * | * | d | * | b | 5 |
| Tehranian [59] | * | b | b | * | * | / | d | * | b | 4 |
| Thompson [60] | * | b | c | * | * | / | d | * | * | 5 |
| Wakai [61] | * | b | b | * | * | / | d | * | c | 4 |
| White [62] | * | * | * | * | * | / | d | * | * | 7 |
| Yan [63] | * | * | b | * | * | / | d | * | * | 6 |
| Eaker [64] | * | * | * | * | * | / | * | * | * | 8 |
| Randi [65] | * | * | b | * | * | * | d | * | * | 7 |

**Selection**

1) Is the case definition adequate?

a) yes, with independent validation (e.g. >1 person/record/time/process to extract information, or reference to primary record source such as medical/hospital/ pathological records) ***** ****

b) record linkage (e.g. ICD codes in database) with no reference to primary record, or self-report with no reference to primary record

c) no description

2) Representativeness of the cases

a) consecutive or obviously representative series of cases (i.e. all eligible cases of breast cancer over a defined period of time, all cases in a defined catchment area, all cases in a defined hospital or clinic, group of hospitals, health maintenance organisation, or an appropriate sample of those cases) *****

b) potential for selection biases or not stated

3) Selection of Controls

a) community controls (i.e. same community as cases and would be cases if had outcome) ***** ****

b) hospital controls, within same community as cases (i.e. not another city) but derived from a hospitalised population

c) no description

4) Definition of Controls

a) no history of breast cancer***** ****

b) no description of source

**Comparability**

1) Comparability of cases and controls on the basis of the design or analysis

a) study controls for age***** ****

b) study also controls for at least three of the following known breast cancer risk factors: family history of BC, parity, OC/HRT usage, age at first and last birth, number of live birth/abortion/miscarriages, birth interval, breastfeeding, lifelong menstrual pattern, menopausal status, age at menarche/menopause, history of BBD, Edu, BMI, alcohol intake, smoking******

**Exposure**

1) Ascertainment of exposure

a) secure record (e.g. regularly-updated marriage registry records) *****

b) structured questionnaire with details on timing of potential changes of marital status***** ****

c) interview not blinded to case/control status

d) written self-report or medical record only

e) no description

2) Same method of ascertainment for cases and controls

a) yes***** ****

b) no

3) Non-Response rate

a) same rate for both groups***** ****

b) non respondents described

c) rate different and no designation
