# Supplementary material for: Does marital status correlate with the female breast cancer risk? A systematic review and meta-analysis of observational studies
Source: PLoS One. 2020 Mar 5;15(3):e0229899. doi: 10.1371/journal.pone.0229899 (PMC7058335; doi:10.1371/journal.pone.0229899)
Supplement: S2 File — (DOCX) [file pone.0229899.s007.docx]

**Table 1. Meta-regression in the multivariate model for unmarried versus married women**

|  | **OR** | **95% CI** | | **P-value** |
| --- | --- | --- | --- | --- |
| Controls type | 0.056 | -0.277 | 0.389 | 0.734 |
| Publication year | -0.006 | -0.024 | 0.012 | 0.501 |
| Adjustment level | 0.056 | -0.252 | 0.363 | 0.715 |
| Geographic region | 0.501 | 0.005 | 0.158 | 0.005 |

OR: Odds Ratio; 95% CI: 95% Confidence Interval

**Table 2. Meta-regression in the univariate model for unmarried versus married women**

|  | **OR** | **95% CI** | | **P-value** |
| --- | --- | --- | --- | --- |
| Controls type | -0.028 | -0.368 | 0.312 | 0.867 |
| Publication year | 0.004 | -0.013 | 0.021 | 0.643 |
| Adjustment level | 0.057 | -0.280 | 0.394 | 0.734 |
| Geographic region | 0.427 | 0.141 | 0.713 | 0.004 |

OR: Odds Ratio; 95% CI: 95% Confidence Interval

**Table 3. Meta-regression in the univariate model for divorced versus married women**

|  | **OR** | **95% CI** | | **P-value** |
| --- | --- | --- | --- | --- |
| Controls type | -0.418 | -0.985 | 0.148 | 0.135 |
| Publication year | 0.029 | 0.005 | 0.0535 | 0.024 |
| Adjustment level | 0.243 | -0.363 | 0.850 | 0.402 |
| Geographic region | 0.704 | 0.147 | 1.261 | 0.017 |

OR: Odds Ratio; 95% CI: 95% Confidence Interval
