# Supplementary material for: Does marital status correlate with the female breast cancer risk? A systematic review and meta-analysis of observational studies
Source: PLoS One. 2020 Mar 5;15(3):e0229899. doi: 10.1371/journal.pone.0229899 (PMC7058335; doi:10.1371/journal.pone.0229899)
Supplement: S3 File — (DOCX) [file pone.0229899.s008.docx]

**
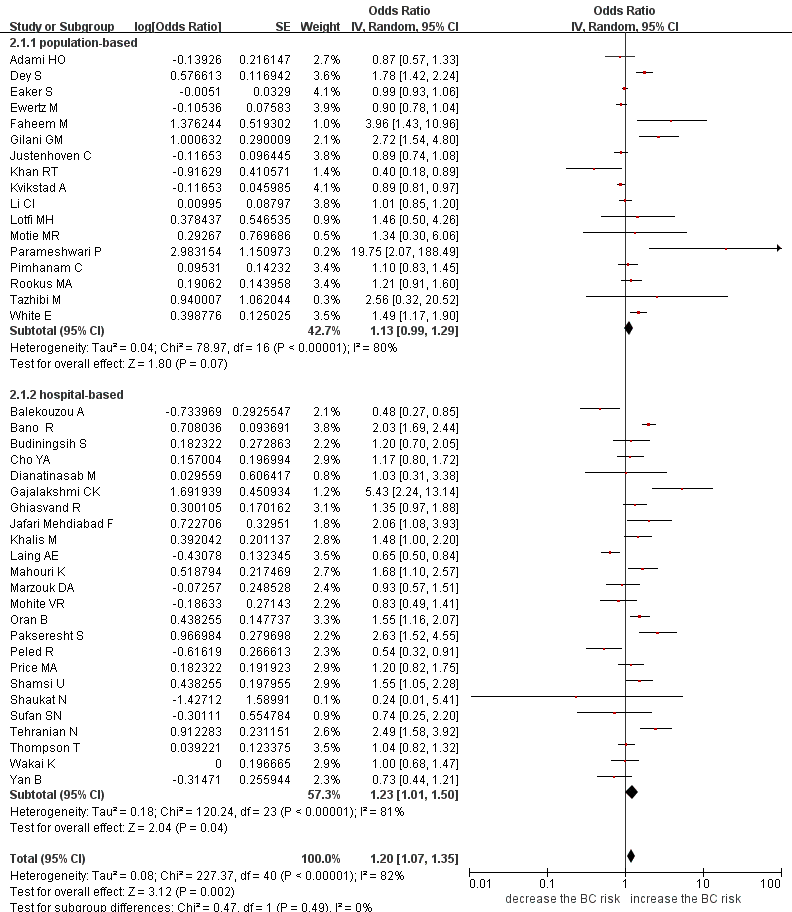
**

**Fig 1.** **Forest Plot of the Breast Cancer Risk among Unmarried Women versus Married Women Stratified by Control Type.**

**
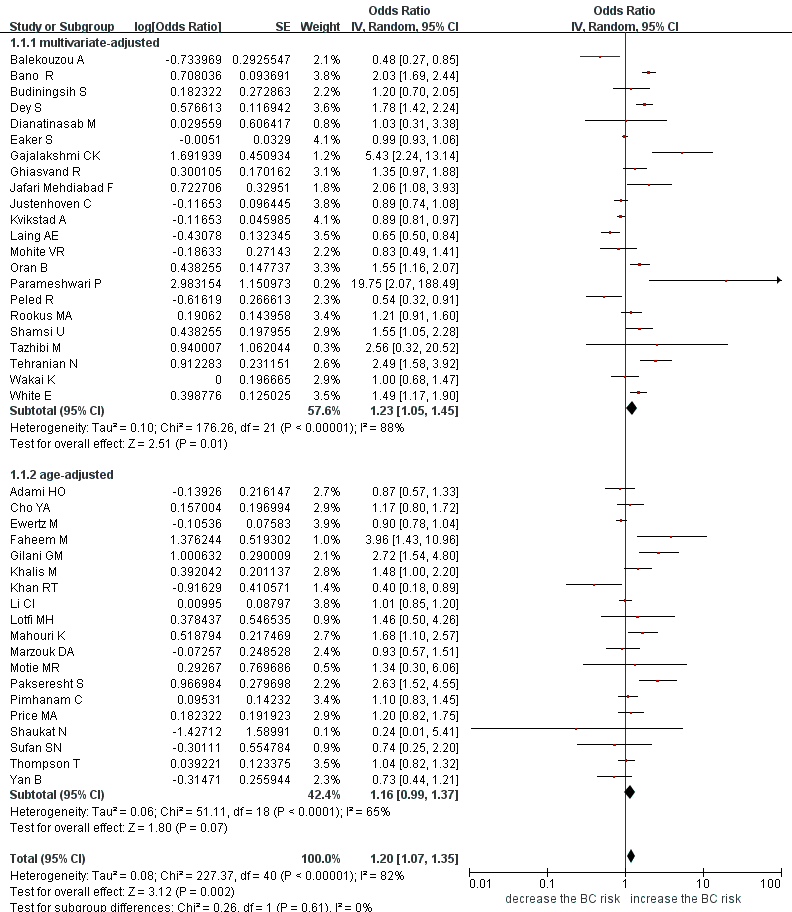
**

**Fig 2.** **Forest Plot of the Breast Cancer Risk among Unmarried Women versus Married Women Stratified by Adjustment Level.**

**
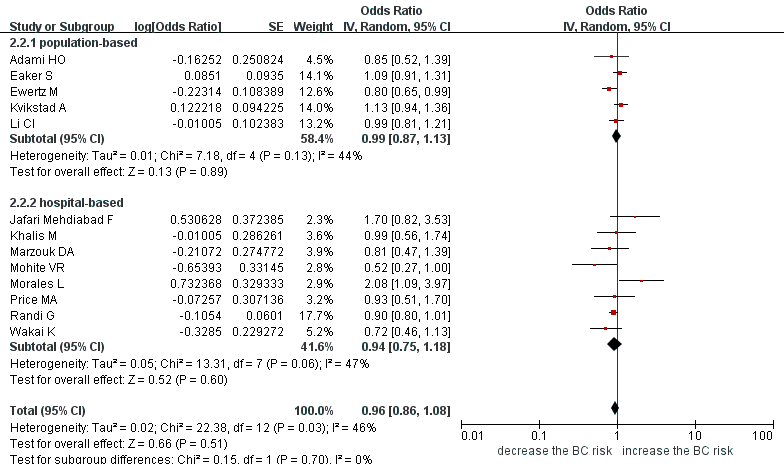
**

**Fig 3.** **Forest Plot of the Breast Cancer Risk among Widowed Women versus Married Women Stratified by Control Type.**

**
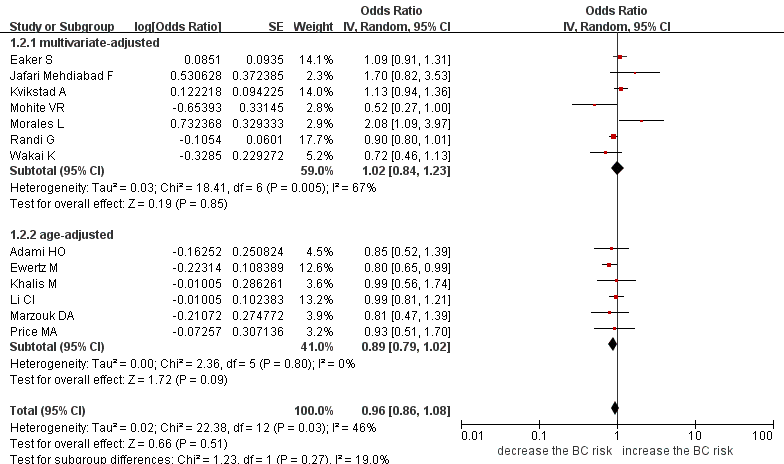
**

**Fig 4.** **Forest Plot of the Breast Cancer Risk among Widowed Women versus Married Women Stratified by Adjustment Level.**

**
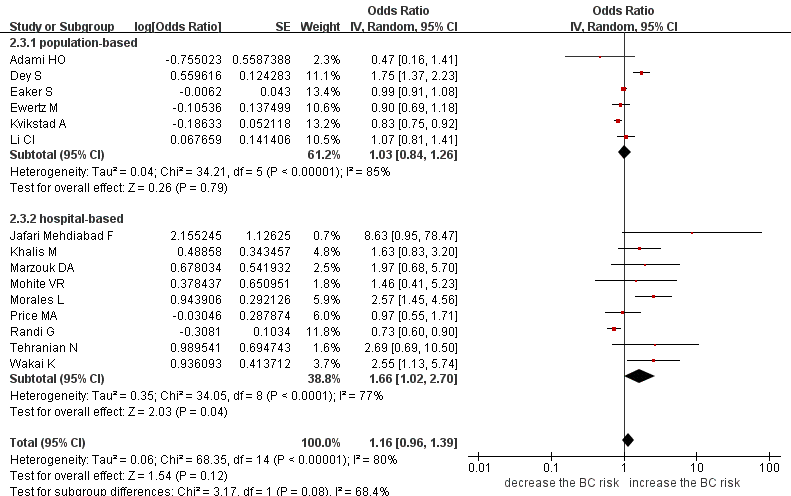
**

**Fig 5.** **Forest Plot of the Breast Cancer Risk among Divorced Women versus Married Women Stratified by Control Type.**

**
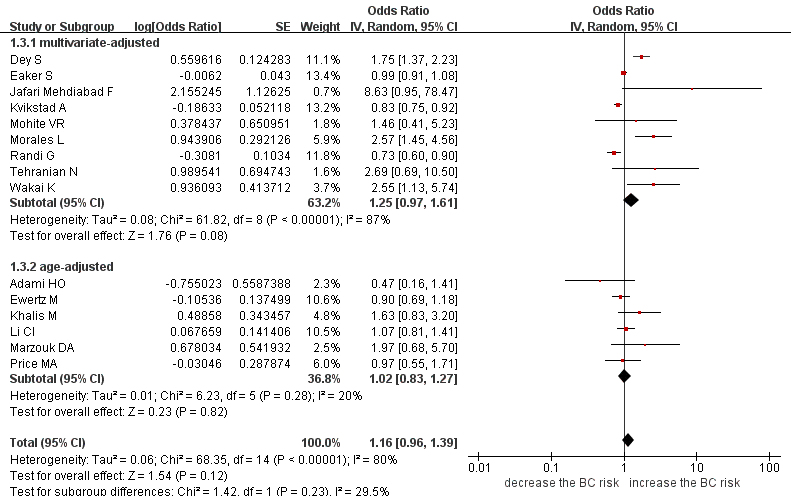
**

**Fig 6.** **Forest Plot of the Breast Cancer Risk among Divorced Women versus Married Women Stratified by Adjustment Level.**

**

**

**Fig 7.** **Forest Plot of the Breast Cancer Risk among Lifelong Single Women versus Married Women Stratified by Control Type.**

(A) Forest plot of cohort studies. (B) Forest plot of case-control studies.

**

**

**Fig 8.** **Forest Plot of the Breast Cancer Risk among Lifelong Single Women versus Married Women Stratified by Adjustment Level.**

(A) Forest plot of cohort studies. (B) Forest plot of case-control studies.
